# The Electrochemical Detection of Ochratoxin A in Apple Juice via MnCO_3_ Nanostructures Incorporated into Carbon Fibers Containing a Molecularly Imprinting Polymer

**DOI:** 10.3390/bios13080760

**Published:** 2023-07-26

**Authors:** Müge Mavioğlu Kaya, Haci Ahmet Deveci, İnan Kaya, Necip Atar, Mehmet Lütfi Yola

**Affiliations:** 1 Department of Molecular Biology and Genetic, Faculty of Arts and Sciences, Kafkas University, Kars 36000, Turkey; m.mavioglu@kafkas.edu.tr; 2 Department of Nutrition and Dietetics, Faculty of Health Sciences, Gaziantep University, Gaziantep 27000, Turkey; h_ahmet_deveci@gantep.edu.tr; 3 Department of Biology, Faculty of Arts and Sciences, Kafkas University, Kars 36000, Turkey; inankaya@kafkas.edu.tr; 4 Department of Chemical Engineering, Faculty of Engineering, Pamukkale University, Denizli 20000, Turkey; natar@pau.edu.tr; 5 Department of Nutrition and Dietetics, Faculty of Health Sciences, Hasan Kalyoncu University, Gaziantep 27000, Turkey

**Keywords:** ochratoxin A, molecularly imprinting, nanocomposite, food analysis

## Abstract

A novel electrochemical sensor based on MnCO_3_ nanostructures incorporated into carbon fibers (MnCO_3_NS/CF), including a molecularly imprinting polymer (MIP), was developed for the determination of Ochratoxin A (OTA). In this study, a sensitive and selective sensor design for OTA detection was successfully performed by utilizing the selectivity and catalysis properties of MIP and the synthesized MnCO_3_NS/CF material at the same time. MnCO_3_ nanostructures incorporated into carbon fibers were first characterized by using various analytical techniques. The sensor revealed a linearity towards OTA in the range of 1.0 × 10^−11^–1.0 × 10^−9^ mol L^−1^ with a detection limit (LOD) of 2.0 × 10^−12^ mol L^−1^. The improved electrochemical signal strategy was achieved by high electrical conductivity on the electrode surface, providing fast electron transportation. In particular, the analysis process could be finished in less than 5.0 min without complex and expensive equipment. Lastly, the molecular imprinted electrochemical sensor also revealed superior stability, repeatability and reproducibility.

## 1. Introduction 

The need for food by people is growing parallel with the increasing human population in the world. People may face serious health problems after exposure to mycotoxin contamination due to the storage conditions of large amounts of food or food raw materials produced for mycotoxin growth. Toxic compounds synthesized as a result of the secondary metabolism of mold species are called “mycotoxins”. Mycotoxins are considered to be very significant natural toxins and are one of the important problems that need to be controlled, as they can be found in all areas, can develop in many foods and feed materials and form toxins, and can be transmitted through nutrition, inhalation and skin contact [1]. OTA, which is one of the mycotoxins produced by certain species of *Aspergillus* and *Penicillium* genus, is structurally an isocoumarin derivative. OTA is a colorless, crystalline compound, soluble in sodium bicarbonate solutions, recrystallized with weak xylene, with low water solubility and acidic properties. It is a fungal compound that threatens the health of people in every climate due to its greater chemically stable and half-life compared to other derivatives and its immunosuppressant properties that affect organs, such as the liver, kidney and thymus, including carcinogen [2]. OTA can also affect many parts of the human body, such as the central nervous system and protein synthesis. Moreover, OTA inhibits the enzyme by competing with the t-RNA synthetase enzyme, which is responsible for protein synthesis, and causes protein synthesis to stop. According to the results of various experimental studies on animals, the amount of OTA, which the human body can tolerate Daily, is between 0.2 and 4.2 ng kg^−1^. Different analysis methods have been developed to control the toxic effects of OTA and not exceed both local and global scientific limits. The quality and future of OTA analysis methods in samples also depend on sensitivity, selectivity, time and cost.

In the literature, the chromatography [3], fluorimetric method [4] and surface-enhanced Raman spectroscopy [5] were developed for OTA detection in real samples. Although SERS analysis and fluorescence spectrometry are useful compared to other tests, it is reported that the stability and accuracy of these tests should be further optimized [6]. However, it can be said that analyzes are not carried out with an environmentalist approach since these methods involve excessive chemical consumption. Thus, the need for more effective, environmentally friendly and rapid analysis techniques, which minimize the above-mentioned reasons, still maintains its importance. Electrochemical techniques are the methods in which the electrode-solvent interaction is maximized, and the optimum analysis results are naturally obtained in the fastest way [7,8]. Especially there have been studies in which voltammetric techniques have been used intensively to ensure food safety [9]. Thanks to these techniques, the possible harmful effects of the pesticides, such as mycotoxins, are predicted in advance by making nutrient analyzes instantly and quickly. In conclusion, thanks to the fast signal acquisition capability of voltammetric methods based on aptasensor [10,11,12,13], the analysis of the target molecules, such as pesticides, is performed instantly, thus ensuring safer food consumption.

Transition metal carbonates have started to attract significant attention in the electrochemical applications [14,15]. In particular, MnCO_3_ nanostructures have been considered as a nanomaterial with high electrochemical application [16,17]. In addition, MnCO_3_ nanostructures show important advantages containing non-toxicity, simple preparation with high yield and probability of being abundant on earth. In literature, MnCO_3_@rGO composite was prepared by hydrothermal method and its capacity was obtained as 857 mAh g^−1^ after a certain scan cycle, providing excellent electrochemical features of MnCO_3_ nanostructures [18]. Nonetheless, its limited electrical conductivity prevents further electrochemical applications. The formation of MnCO_3_ nanostructure composites, including carbon fiber, carbon nanotube and graphene oxide, causes the enhancement of electrochemical conductivity and activity. Especially, MnCO_3_ nanostructures incorporated into carbon fibers may be a better choice in comparison with carbon-based nanomaterials owing to the important application of the MnCO_3_ nanostructure [17]. Carbon fibers are 4.5 times lighter than steel material and three times more durable, increasing their usage areas. Carbon fibers with a long life are preferred because of their lightness in terms of structure. In addition, it provides a visual advantage as it does not have a problem such as oxidation. They have a lower density than steel, making them a unique material for applications requiring a high weight ratio [19].

MIPs contain active sites that complete the three-dimensional structure of the substrate on the “Key-Lock” model so that the substrate interacts with the active sites that complete its specific structure, just like the key. Molecular imprinting is generally the process of synthesizing polymers with specific recognition sites specific to the template molecule. There are essential components required for the synthesis process: (i) template molecule or molecule to be imprinted, (ii) monomer, (iii) crosslinker, (iv) initiator. After the polymerization process, the analyte molecule is removed from the polymer by washing in order to form spaces in the structure to replace the analyte molecule. The formed cavities recognize the size, structure and chemical properties of the removed analyte molecule and provide binding of the analyte molecule under appropriate conditions [20,21]. In the following processes, when a solution containing the analyte molecule is applied to the imprinted polymer, it will recognize the analyte molecule thanks to its active recognition sites, and, thus, the analyte molecule will be re-attached to the polymer [22]. To prepare a molecularly imprinted polymer, two different approaches are used, covalent and non-covalent, depending on the interaction between the functional monomer and the template molecule [22,23]. In covalent imprinting, covalent bonding occurs first between the functional monomer and the template molecule. In order to form MIPs after polymerization, the covalent bonds formed are broken and removed from the polymer [24]. In this imprinting, functional monomers, which can interact non-covalently with the template molecule, are used. The interaction between the functional monomer and the template molecule occurs through non-covalent interactions, such as hydrogen bonding and van der Waals interactions. When the polymerization is completed, the template molecule is removed from the polymer with the help of the solvent [25]. Pyrrole monomer is one of the most widely used conductive polymers in MIPs-based electrochemical sensor applications because of its high conductivity, high stability in a wide range of pH, electropolymerization efficiency and physical/chemical inertness [26,27].

There are some MIP-based analytical methods developed for OTA analysis in the literature. For instance, MIP-based magnetic photonic crystal microspheres for OTA analysis were prepared, and a linear detection range of 5–20,000 ng mL^−1^ and a LOD of 0.675 ng mL^−1^ were obtained with a good recovery [28]. In addition, the chromatographic detection based on microfiber graphene/nanofibers, including MIP, was completed for OTA determination in milk beverages and a linear detection range of 0.2–40.0 µg L^−1^ was determined with good repeatability [29]. In another study, OTA imprinted nanoMIPs were developed by using a solid-phase polymerization technique, including phenylalanine derivative as a monomer. The results showed that the high selectivity was accomplished [30]. In another study, a MIP-assisted sample clean-up method was developed to remove OTA from wine samples. This method was based on a two-dimensional solid-phase extraction on C18-silica, and the developed method showed a LOD of 0.01 ng mL^−1^ [31]. The oligo(ethylene glycol) monomethyl ether methacrylate-based MIPs was also synthesized by atom transfer radical polymerization for OTA removal, and the values of recovery were obtained as 97.1–97.4% [32]. Finally, a ratiometric electrochemical sensor based on magnetic MIP was presented with high reproducibility and a linear detection range of 0.5–15.0 µmol L^−1^ and a LOD of 14.0 nmol L^−1^ was obtained [33]. 

This paper provides a rapid and selective OTA detection method via a molecularly imprinted sensor based on MnCO_3_ nanostructures incorporated into carbon fibers. Thanks to the high electrical conductivity caused by MnCO_3_ nanostructures incorporated into carbon fibers on the electrode surface, a sensitive OTA analysis was performed with high selectivity. Hence, a seminal study has been presented to the world of literature in terms of safer food consumption by developing a novel OTA imprinted sensor.

## 2. Experimental

### 2.1. Chemicals and Apparatus

OTA, ochratoxin B (OTB), aflatoxin B_1_ (AFB1), aflatoxin B_2_ (AFB2), citrinin (CIT), catechol (CAT), resveratrol (RES), KMnO_4_ and pyrrole (Py) monomer were maintained by Sigma-Aldrich. Phosphate-buffered saline (0.1 mol L^−1^, pH 7.0, PBS) of supporting electrolytes was utilized for electrochemical measurements. 

ZEISS EVO 50 SEM (Tokyo, Japan), JEOL 2100 TEM (Tokyo, Japan), a Rikagu Miniflex X-ray diffractometer using mono-chromatic CuKα (new 6th-generation general purpose benchtop system, Tokyo, Japan) and a PHI 5000 Versa Probe type X-ray photoelectron spectrometer (Tokyo, Japan) techniques were applied for the determination of structural analysis of MnCO_3_NS and MnCO_3_NS/CF composite. Gamry Reference 600 workstation (Warminster, PA, USA) was also performed for the electrochemical investigations by using DPV, EIS, and CV. 

### 2.2. Production of MnCO_3_NS and MnCO_3_NS/CF Composite

The hydrothermal technique was utilized for the preparation of the MnCO_3_NS/CF composite. The commercial carbon fiber cloth (pyrolytically stripped, platelets(conical), >98% carbon basis) was used as a source for the carbon fibers and maintained by Sigma-Aldrich. Before MnCO_3_ growth, the commercial carbon fiber (CF) cloth interacted with 50:50 (*v*/*v*) ethanol and acetonitrile solution under strong sonication for 20 min. Then, CF was activated in concentrated HNO_3_ (50.0%) for 45 min. After that, the solution of KMnO_4_ (50.0 mmol L^−1^) and starch (2.0 g) in ultra-pure water (50.0 mL) was prepared, and this solution was subjected to stirring treatment for 45 min. Then, the solution and CF piece (3.0 cm × 3.0 cm) were transferred to a Teflon-lined hydrothermal autoclave (Techinstro Industries, Maharashtra, India) and subjected to the heating treatment at 180 °C for 10 h. The product MnCO_3_NS/CF composite was washed with ultra-pure water two times and dried at 100 °C. In addition, MnCO_3_NS was prepared by the same procedure without CF [17].

### 2.3. Production of MnCO_3_NS/CF Modified Glassy Carbon Electrode (MnCO_3_NS/CF/GCE)

The procedure of surface cleaning of GCE electrodes was performed according to our previous paper [34]. The GCE was modified with MnCO_3_NS/CF composite solution (40.0 μL, 0.5 mg mL^−1^) and an IR lamp was utilized to remove the solvent for 20 min, providing MnCO_3_NS/CF/GCE. MnCO3NS-modified GCE surfaces (MnCO_3_NS/GCE) were formed by the method described above.

### 2.4. OTA Imprinted Sensor and OTA Removal 

**Scheme 1:**
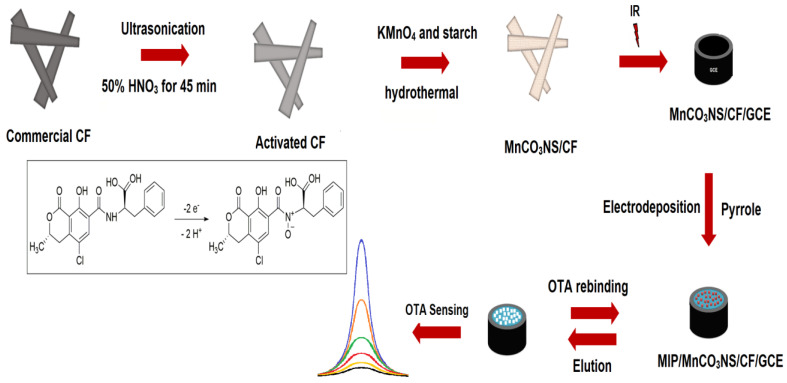
Preparation of MnCO_3_NS/CF composite and OTA imprinted electrode.

Firstly, 25.0 mmol L^−1^ OTA solution, including 100.0 mmol L^−1^ Py was prepared into the cell for the development of OTA imprinted polymer on MnCO_3_NS/CF/GCE. Then, 20 continuous cycles were scanned on MnCO_3_NS/CF/GCE in +0.00/+1.00 V range, and the current responses at +0.70 V were constantly followed to produce OTA imprinted MnCO_3_NS/CF/GCE (MIP/MnCO_3_NS/CF/GCE) (Scheme 1) [35]. Then, the template molecule (OTA) was removed by using NaCl solution (10.0 mL, 1.0 mol L^−1^). The described method was utilized for the development of OTA non-imprinted MnCO_3_NS/CF/GCE (NIP/MnCO_3_NS/CF/GCE) to indicate the imprinting selectivity. 

### 2.5. Sample Preparation

Apple juice samples taken from the local supermarket were first kept in the refrigerator at +4 °C for 1 day and then transferred to a test tube. After adding ethanol (5.0 mL) to this test tube, it was exposed to a shake bath system for 15 min. After the centrifugation for 5 min at 1000 rpm, the upper phase was spiked with a fine syringe [36]. Then, the upper phase was diluted with 0.1 M PBS (3.0 mL, pH 7.0) and transported into the electrochemical cell for OTA analysis. MIP/MnCO_3_NS/CF/GCE, platinum wire and Ag/AgCl/KCl_(sat)_ were used for the working electrode, counter electrode and reference electrode, respectively, for the analysis. Analytical applications were carried out from the calibration graph obtained by plotting the current values of the peaks occurring at +0.5 V in the +0.2/+1.0 V potential range against the OTA concentration. 

## 3. Results and Discussion 

### 3.1. Characterizations of MnCO_3_NS/CF

**Figure 1:**
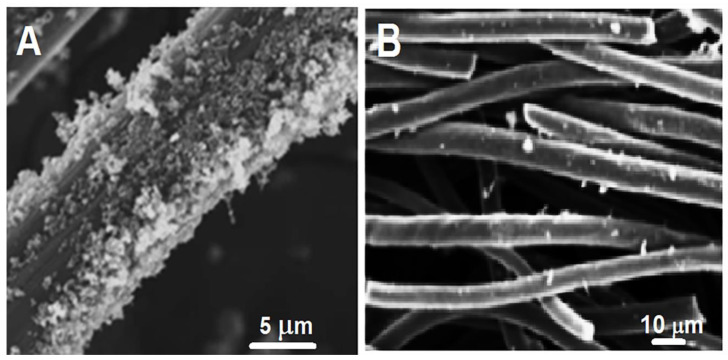
(**A**,**B**) SEM images of MnCO_3_NS/CF composite at different magnifications.

After the ultrasonication treatment of commercial CF in HNO_3_ (50%) for 45 min, MnCO_3_NS was interconnected onto the CF surface by hydrothermal process. The surface characterizations of CF and MnCO_3_NS were explored by SEM (Figure S1). According to Figure S1A, the diameter of the smooth carbon fiber was calculated as about 7–8 µm, and the diameter of pure MnCO_3_NS was obtained as about 3–4 µm (Figure S1B). After the preparation of MnCO_3_NS interconnected onto the CF surface by hydrothermal method, the granular CF formation occurred via MnCO_3_NS interconnection (Figure 1A,B). According to Figure 1, the diameter of MnCO_3_NS/CF was calculated to be about 9–10 µm, indicating the successful preparation of MnCO_3_NS on the CF surface, including interconnected interaction. 

TEM measurements were performed for the determination of the elemental composition of MnCO_3_NS (Figure S2). According to Figure S2A, the size of MnCO_3_NS was obtained as about 50–51 nm. This result was confirmed in harmony with SEM measurements. In addition, unclear crystalline fringes (Figure S2B) verified the non-crystallization of MnCO_3_NS. The presence of C, Mn, and O elements was verified on the EDX image (Figure S2C), providing the successful preparation of MnCO_3_NS/CF nanocomposite and the specific surface area of MnCO_3_NS/CF nanocomposite, including interconnected interaction. This specific surface area also indicated efficient contact with electrolyte solution and suggested the superior electrochemical activity [17]. 

**Figure 2:**
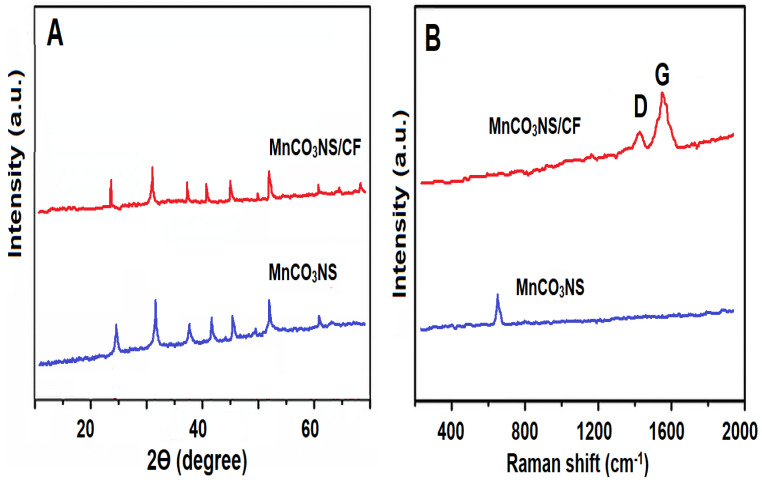
(**A**) XRD patterns of MnCO_3_NS and MnCO_3_NS/CF composite (Six obvious XRD peaks at 24.08°, 31.79°, 37.79°, 41.93°, 45.07° and 51.36° corresponded to (012), (104), (110), (113), and (204) planes, respectively) and (**B**) Raman spectra of MnCO_3_NS and MnCO_3_NS/CF composite (G band is the in-plane vibrations of sp^2^ hybridization of carbon atoms and D band is known as the irregularity band or the defect band).

The crystal structures of MnCO_3_NS and MnCO_3_NS/CF composite were investigated by XRD (Figure 2A). Six obvious XRD peaks at 24.08°, 31.79°, 37.79°, 41.93°, 45.07° and 51.36° corresponded to (012), (104), (110), (113), and (204) planes, respectively. XRD peak at 24.08° was attributed to the carbon phase, and there were no impurity peaks, indicating that the successful preparations of MnCO_3_NS and MnCO_3_NS/CF composite were performed in this work. In addition, Raman spectra (Figure 2B) were recorded for the structural investigation of MnCO_3_NS and MnCO_3_NS/CF composite. Three obvious Raman peaks at 320 cm^−1^, 373 cm^−1^ and 660 cm^−1^ were attributed to the specific peaks of MnCO_3_NS. The Raman peaks at 320 cm^−1^ and 373 cm^−1^ corresponded to the translational and librational modes. The peak at 660 cm^−1^ also corresponded to the in-plane bend mode [15]. Finally, two Raman peaks at 1392 cm^−1^ and 1591 cm^−1^ were attributed to the D band and G band of CF. Thus, XRD and the Raman results confirmed the successful preparation of MnCO_3_NS on the CF surface. 

**Figure 3:**
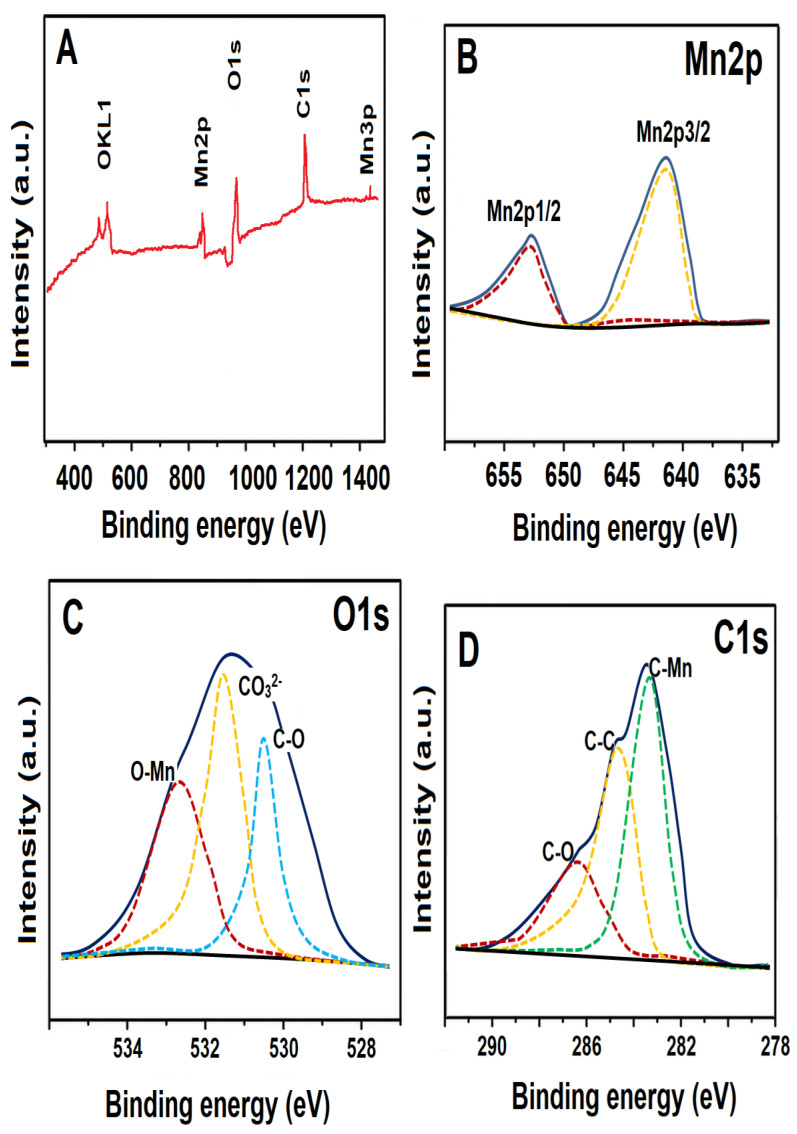
(**A**) XPS survey spectrum of MnCO_3_NS/CF composite and high-resolution spectra of (**B**) Mn2p (red line: Mn2p^1/2^, yellow line: Mn2p^3/2^) (**C**) O1s (red line: O-Mn, yellow line: CO_3_^2−^, blue line: C-O) and (**D**) C1s (red line: C-O, yellow line: C-C, green line: C-Mn).

Mn2p, O1s and C1s high-resolution XPS spectrums of MnCO_3_NS/CF composite were given in Figure 3. According to the survey spectrum of Figure 3A, the presence of manganese, oxygen and carbon elements verified MnCO_3_NS/CF composite. The XPS peaks at 653.08 eV and 640.39 eV on Mn2p spectra (Figure 3B) were attributed to Mn2p1/2 and Mn2p3/2, respectively [37]. In addition, three XPS peaks at 532.71 eV, 531.09 eV and 530.46 eV corresponded to O-Mn, CO_3_^2−^ and C-O bonds, respectively (Figure 3C). On the C1s spectrum (Figure 3D), three XPS peaks at 286.13 eV, 284.69 eV and 283.94 eV corresponded to C-O, C-C and C-Mn, respectively. It was concluded that the C-Mn bond resulted from the interaction between MnCO_3_ and CF. Hence, MnCO_3_NS was bridged to CF via C-Mn bonds, providing chemical stability during the electrochemical sensor application [38,39].

### 3.2. Electrochemical Characterizations of MnCO_3_NS and MnCO_3_NS/CF Composite Modified Electrodes

**Figure 4:**
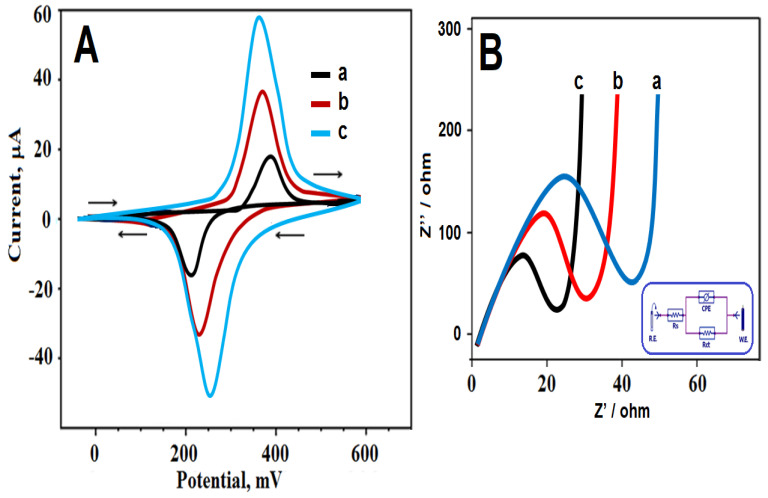
(**A**) CV curves (arrow indicates potential scanning direction) and (**B**) EIS responses at (a) bare GCE, (b) MnCO_3_NS/GCE, (c) MnCO_3_NS/CF/GCE.

CV and EIS measurements were obtained to demonstrate the electrochemical performances of MnCO_3_NS/GCE and MnCO_3_NS/CF/GCE in 5.0 mmol L^−1^ [Fe(CN)_6_]^3−/4−^ (Figure 4). Electrochemical current signals at +0.400 and +0.200 V appeared at bare GCE (curve a of Figure 4A). However, the more prominent electrochemical performances were obtained at MnCO_3_NS/GCE (curve b of Figure 4A) due to the large battery capacity of the MnCO_3_NS [16,40]. After the preparation of MnCO_3_NS interconnected onto the CF surface, the more easy electron transfer occurred on MnCO_3_NS/CF/GCE, and the obvious electrochemical signals were observed in comparison with MnCO_3_NS/GCE (curve c of Figure 4A) [17]. If we want to examine the electrochemical oxidation process, the oxidation of the OTA molecule occurred based on a mechanism involving 2 protons and 2 electrons through the amine group (Scheme 1) [41]. The number of the transferred electrons was calculated by using Laviron’s Equation (1) (See Supplementary Data for the explanations of terms):

E_p_ = E^0^ + [RT/(1 − α)nF] Inν (1)

In addition, when CV anodic peak currents were plotted against the square root of the scan rate (10–1000 mV s^−1^) (R^2^ = 0.9991), the obtained linearity indicated that the oxidation phenomenon was diffusion-controlled [42]. 

EIS measurements were applied to confirm the CV results (Figure 4B). Experimental data fitting to the Randles circuit for MnCO_3_NS/CF/GCE were shown on the inset of Figure 4B, including the charge transfer resistance (R_ct_), the solution resistance (R_s_) and the constant phase element (CPE). R_ct_ values were studied to be 45 ohm for bare GCE, 35 ohm for MnCO_3_NS/GCE, and 25 ohm for MnCO_3_NS/CF/GCE, confirming the accuracy of CV results.

### 3.3. Fabrication of OTA Imprinted Polymer on MnCO_3_NS/CF/GCE

**Figure 5:**
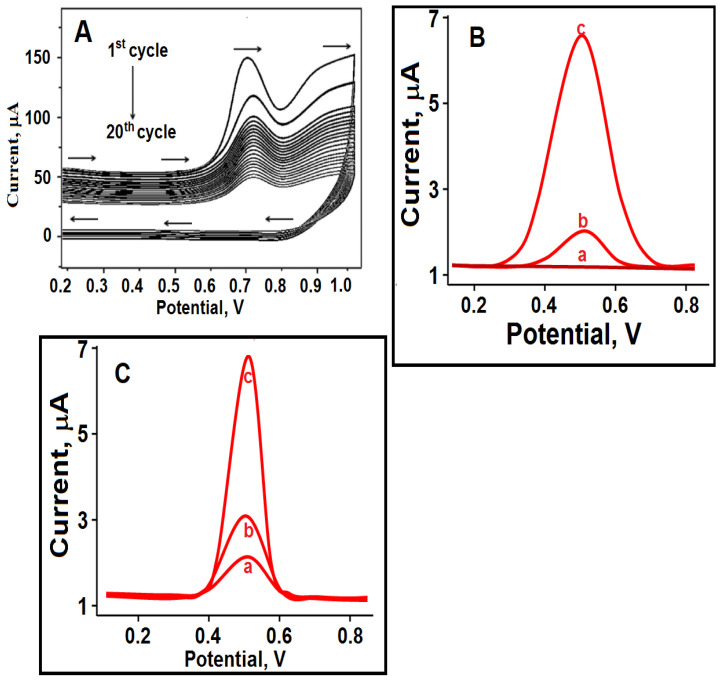
(**A**) 100.0 mmol L^−1^ Py polymerization containing 25.0 mmol L^−1^ OTA on MnCO_3_NS/CF/GCE (Scan rate: 100 mV s^−1^) (arrow indicates potential scanning direction), (**B**) Differential pulse voltammograms (DPVs) of MIP and NIP electrodes: (a) in presence of PBS solution, (b) NIP/MnCO_3_NS/CF/GCE in presence of 0.5 nmol L^−1^ OTA and (c) MIP/MnCO_3_NS/CF/GCE in presence of 0.5 nmol L^−1^ OTA, (**C**) DPVs of different molecularly imprinting electrodes (a) MIP/bare GCE, (b) MIP/MnCO_3_NS/GCE and (c) MIP/MnCO_3_NS/CF/GCE.

According to the increase in the number of scans, the permanent decrease in electrochemical signals proved that OTA imprinted polymers were formed on MnCO_3_NS/CF/GCE (Figure 5A). Electrochemical measurements were made using MIP and NIP electrodes in the presence of 0.5 nmol L^−1^ OTA and without OTA to show that molecularly imprinted polymers bring a high selectivity to the target molecule. As expected, no current signals were observed on MIP/MnCO_3_NS/CF/GCE in the presence of pH 7.0, PBS solution (Curve a of Figure 5B). In comparison with MIP (curve c of Figure 5B) and NIP (curve b of Figure 5B) electrodes, it was seen that OTA imprinted molecularly imprinted polymers provided a more selective sensor affinity towards OTA molecules. In addition, three OTA imprinted electrochemical electrodes were prepared and applied to 0.5 nmol L^−1^ OTA in the presence of pH 7.0, PBS solution (Figure 5C). These results, which were in agreement with Figure 5B, prove that the MIP/MnCO_3_NS/CF/GCE electrode can be used for future studies.

### 3.4. Optimization Studies

#### 3.4.1. pH Effect

**Figure 6:**
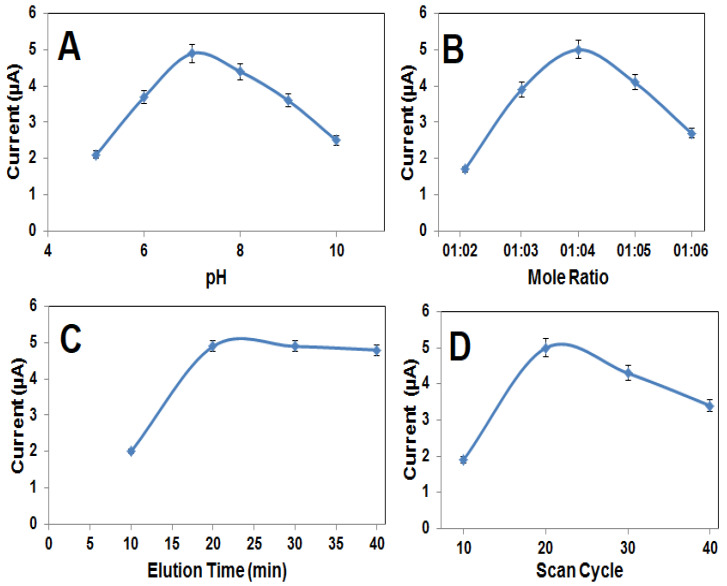
Effect of (**A**) pH, (**B**) mole ratio, (**C**) desorption time, (**D**) scan cycle on signals of DPVs (in the presence of 0.5 nmol L^−1^ OTA) (*n* = 6).

The pH of the working environment is the most important parameter affecting the signal strength of the working electrode in electrochemical sensor studies. When Figure 6A was examined, while the working electrode produced maximum signal up to pH 7.0, these signals decreased after pH 7.0. Thus, 7.0 was chosen as the ideal pH.

#### 3.4.2. Mole Ratio OTA to Py Monomer Effect

In molecularly imprinted electrochemical sensor studies, the stoichiometric ratio between the analyte and the monomer significantly affects the sensitivity of the method. When the monomer ratio is low, the number of binding sites on the electrode surface is low. Thus, 100.0 mmol L^−1^ Py and 25.0 mmol L^−1^ OTA were used as monomer and target molecules (Figure 6B).

#### 3.4.3. Desorption Time Effect

Complete removal of the analyte molecule from the electrode surface is an important factor in fast electrochemical sensor designs. In this study, several desorption times were tried, and it was seen that the analyte molecules were completely removed from the electrode surface at the end of the 20th min (Figure 6C).

#### 3.4.4. Scan Cycle Effect

In electrochemistry-based polymerization techniques, the surface thickness of the working electrode is another significant factor affecting the performance of the sensor. The number of scans must be kept at an optimal level so that non-specific interactions caused by the thick monomer layer on the surface of the working electrode do not occur. We observed that working electrodes with 20 scan numbers produced the highest sensor response when working electrodes prepared with different scan numbers were used (Figure 6D).

### 3.5. Quantification Limit (LOQ) and LOD Values 

**Figure 7:**
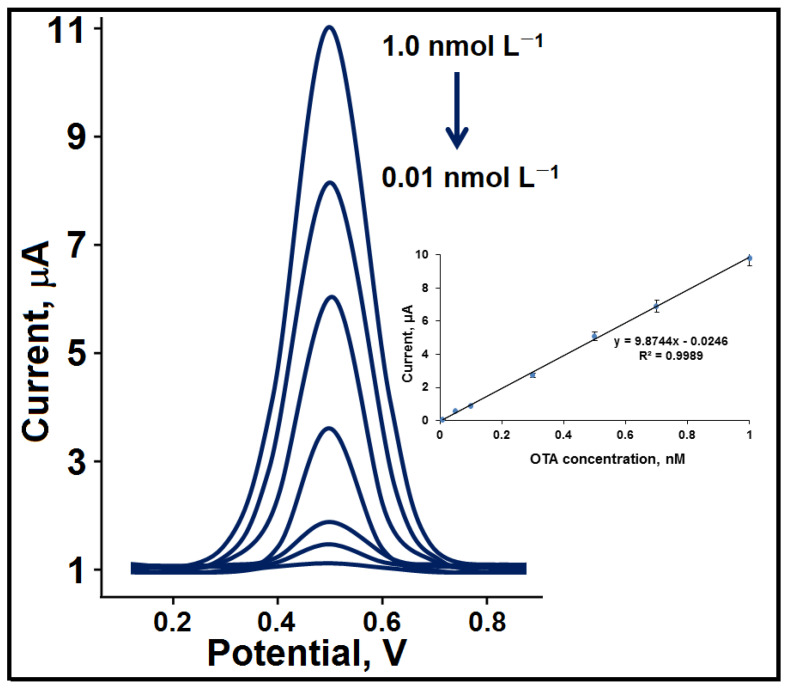
DPVs with different OTA amount at MIP/MnCO_3_NS/CF/GCE in pH 7.0 of PBS. Inset: OTA’s calibration curve (from 0.01 nmol L^−1^ to 1.0 nmol L^−1^).

The linearity range of MIP/MnCO_3_NS/CF/GCE was first formed by using OTA concentration and DPV signals. Thus, y (µA) = 9.8744x (C_OTA_, nmol L^−1^) − 0.0246, (R^2^ = 0.9989) was given in Figure 7 and LOQ, and LOD values were computed as 1.0 × 10^−11^ mol L^−1^, and 2.0 × 10^−12^ mol L^−1^, respectively (See Supplementary Data for the equations). In addition, Table 1 indicates the sensitivity features of MIP/MnCO_3_NS/CF/GCE in comparison with the existing methods for OTA detection. Thus, we presented a more sensitive detection method for OTA in comparison with the reported methods. Since we produced MnCO_3_NS/CF composite by using a hydrothermal process, we developed an environmentally friendly analytical process for OTA detection and zero waste generation occurred during sensor preparation. Due to the high stability, selectivity, repeatability and reproducibility, the OTA imprinted sensor is also utilized as a reference technique for mycotoxin detection. Lastly, we believe that this OTA imprinted sensor will be a pioneer for healthier food consumption.

### 3.6. Recovery Assessment

Recovery experiments were performed in apple juice. According to Table 2, the values close to 100% verified a high recovery of MIP/MnCO_3_NS/CF/GCE.

### 3.7. Selectivity, Stability, Repeatability and Reproducibility Performances of MIP/MnCO_3_NS/CF/GCE

For selectivity experiments of MIP/MnCO_3_NS/CF/GCE, 6 chemical agents with similar chemical features were detected (OTB, AFB1, AFB2, CIT, CAT and RES). The electrochemical signals (µA) were obtained for 1.0 nmol L^−1^ OTA, 100.0 nmol L^−1^ OTB, 100.0 nmol L^−1^ AFB1, 100.0 nmol L^−1^ AFB2, 100.0 nmol L^−1^ CIT, 100.0 nmol L^−1^ CAT and 100.0 nmol L^−1^ RES on MIP/MnCO_3_NS/CF/GCE and NIP/MnCO_3_NS/CF/GCE (Figure S3A,B). These electrochemical signals are given in Table 3, including selectivity coefficient (k) and relative selectivity coefficient (k′) values. It was concluded that MIP/MnCO_3_NS/CF/GCE was 7.92, 9.50, 12.67, 19.00, 31.67 and 47.50 times more selective for OTA than OTB, AFB1, AFB2, CIT, CAT and RES, respectively, owing to specific nano-cavities of OTA. 

The stability test of the OTA imprinted sensor was evaluated for 7 weeks (Figure S4). The observed current response in the seventh week was ~97.19% of the current response in the first week, indicating the high stability of the OTA imprinted electrode. 

The relative standard deviation (RSD) value was obtained as 0.19% for 45 consecutive measurements. Hence, the high repeatability of one MIP/MnCO_3_NS/CF/GCE electrode was confirmed.

Finally, the reproducibility test of 20 different OTA imprinted sensors was investigated, and 20 different OTA imprinted sensors were applied to 0.5 nmol L^−1^ OTA. RSD of 0.77% was obtained for 20 different measurements, providing a high reproducibility.

## 4. Conclusions

In this study, a sensitive and selective molecularly imprinted electrochemical sensor based on MnCO_3_NS/CF was presented for OTA detection. MnCO_3_NS/CF composite improved the electrochemical performances in OTA analysis. This result can be explained by using these reasons: (i) MnCO_3_NS, including interconnected structures, provided high electrical conductivity during the electrochemical process, ensuring rapid electron transportation. (ii) MnCO_3_NS was incorporated into the CF surface through Mn-C bonds, providing electrode stability. According to analytical data, the OTA imprinted electrode indicated linearity (1.0 × 10^−11^–1.0 × 10^−9^ mol L^−1^) with a low LOD of 2.0 × 10^−12^ mol L^−1^, ensuring excellent sensitivity and selectivity. The fact that the developed sensor also has an environmentally friendly feature will lead to the emergence of new perspectives for the detection and analysis of other mycotoxins in the future. In addition, OTA imprinted electrode’s ability to make rapid analysis in a short time can cause more reliable food consumption.

## Figures and Tables

**Table 1:** Comparison of MIP/MnCO_3_NS/CF/GCE with the reported methods for OTA detection.

Material	Linear Range (M, mol L^−1^)	LOD (M, mol L^−1^)	Ref.
Aptasensor	3.6 × 10^−10^–6.9 × 10^−10^	3.6 × 10^−10^	[43]
SERS aptasensor	1.0 × 10^−10^–4.0 × 10^−8^	3.0 × 10^−11^	[44]
Au NPs/PIL-FMNS/CNT-MoS_2_	5.0 × 10^−7^–15.0 × 10^−6^	1.4 × 10^−8^	[33]
Fluorescence quenching/AuNPs	5.0 × 10^−9^–1.0 × 10^−7^	4.8 × 10^−9^	[45]
CuNPs	2.5 × 10^−9^–2.5 × 10^−7^	2.0 × 10^−9^	[46]
g-CNQDs	1.0 × 10^−9^–1.4 × 10^−7^	5.0 × 10^−10^	[47]
MIP-MWCNTs	5.0 × 10^−8^–1.0 × 10^−6^	4.1 × 10^−9^	[48]
NGQDs-Apt	1.0 × 10^−8^–5.0 × 10^−6^	2.3 × 10^−9^	[49]
Graphene oxide	0.0–2.0 × 10^−7^	1.0 × 10^−10^	[50]
Fluorescent aptasensor	0.0–1.0 × 10^−6^	2.0 × 10^−8^	[51]
Colorimetric aptasensor	1.0 × 10^−11^–3.2 × 10^−8^	1.0 × 10^−11^	[52]
MIP/MnCO_3_NS/CF/GCE	1.0 × 10^−11^–1.0 × 10^−9^	2.0 × 10^−12^	This study

**Table 2:** Recovery results of OTA in apple juice (*n* = 6).

Sample	Added OTA (nmol L^−1^)	Found OTA (nmol L^−1^)	* Recovery (%)
Apple Juice	-	0.307 ± 0.001	-
	0.100	0.406 ± 0.003	99.75 ± 0.06
	0.300	0.608 ± 0.004	100.17 ± 0.08
	0.500	0.808 ± 0.002	100.12 ± 0.05

* Recovery = Found OTA, nmol L^−1^/Real OTA, nmol L^−1^.

**Table 3:** k and k′ values of OTA imprinted electrodes (MIP/MnCO_3_NS/CF/GCE and NIP/MnCO_3_NS/CF/GCE).

	MIP		NIP		
	∆*I* (µA)	k	∆*I* (µA)	k	k′
OTA	9.50	-	1.75	-	-
OTB	1.20	7.92	0.50	3.50	2.26
AFB1	1.00	9.50	0.40	4.38	2.17
AFB2	0.75	12.67	0.30	5.83	2.17
CIT	0.50	19.00	0.20	8.75	2.17
CAT	0.30	31.67	0.15	11.67	2.71
RES	0.20	47.50	0.10	17.50	2.71

k = ∆*I*_OTA_/∆*I* _interfering chemical_ and k′ = k_MIP/_k_NIP_.

## Data Availability

The original data in this study are included in this study, and further inquiries can be directed to the corresponding author.

## Supplementary Materials

The following supporting information can be downloaded at: https://www.mdpi.com/article/10.3390/bios13080760/s1, Figure S1: SEM images of (A) CF and (B) MnCO_3_NS; Figure S2: (A) TEM image of MnCO_3_NS, (B) HRTEM image of MnCO_3_NS and (C) EDX spectrum of MnCO_3_NS/CF composite; Figure S3: DPVs of (A) MIP/MnCO_3_NS/CF/GCE and (B) NIP/MnCO_3_NS/CF/GCE in 1.0 nmol L^−1^ OTA, 100.0 nmol L^−1^ OTB, 100.0 nmol L^−1^ AFB1, 100.0 nmol L^−1^ AFB2, 100.0 nmol L^−1^ CIT, 100.0 nmol L^−1^ CAT and 100.0 nmol L^−1^ RES; Figure S4: Stability test of MIP/MnCO_3_NS/CF/GCE including 0.5 nmol L^−1^ OTA (*n* = 6).
