# Supplementary material for: The Electrochemical Detection of Ochratoxin A in Apple Juice via MnCO3 Nanostructures Incorporated into Carbon Fibers Containing a Molecularly Imprinting Polymer
Source: Biosensors (Basel). 2023 Jul 26;13(8):760. doi: 10.3390/bios13080760 (PMC10452824; doi:10.3390/bios13080760)
Supplement: Supplementary file 1 [file biosensors-13-00760-s001.zip › biosensors-2516001-supplementary.pdf]

biosensors

MDPI

Article

The Electrochemical Detection of Ochratoxin A in Apple Juice via $MnCO_3$ Nanostructures Incorporated into Carbon Fibers Containing a Molecularly Imprinting Polymer

Müge Mavioğlu Kaya [1], Haci Ahmet Deveci [2], İnan Kaya [3], Necip Atar [4] and Mehmet Lütfi Yola [5,*]

[1] Department of Molecular Biology and Genetic, Faculty of Arts and Sciences, Kafkas University, Kars 36000, Turkey; m.mavioglu@kafkas.edu.tr
[2] Department of Nutrition and Dietetics, Faculty of Health Sciences, Gaziantep University, Gaziantep 27000, Turkey; h_ahmet_deveci@gantep.edu.tr
[3] Department of Biology, Faculty of Arts and Sciences, Kafkas University, Kars 36000, Turkey; inankaya@kafkas.edu.tr
[4] Department of Chemical Engineering, Faculty of Engineering, Pamukkale University, Denizli 20000, Turkey; natar@pau.edu.tr
[5] Department of Nutrition and Dietetics, Faculty of Health Sciences, Hasan Kalyoncu University, Gaziantep 27000, Turkey
* Correspondence: mlutfi.yola@hku.edu.tr; Tel.: +90-342-211-8080; Fax: +90-342-211-8081

Linearity

LOQ = 10.0 S / m
LOD = 3.3 S / m
S: Standard deviation of the intercept and m*:* Slope of the regression line
Laviron's equation: $E_p = E^0 + [RT/(1 - \alpha)nF]\ \mathrm{In}\nu$
E^0: Formal potential (Volt)
R: Gas constant
T: Temperature (K)
α: Charge transfer coefficient,
F: Faraday constant,
n: Number of the transferred electrons
ν: Scan rate

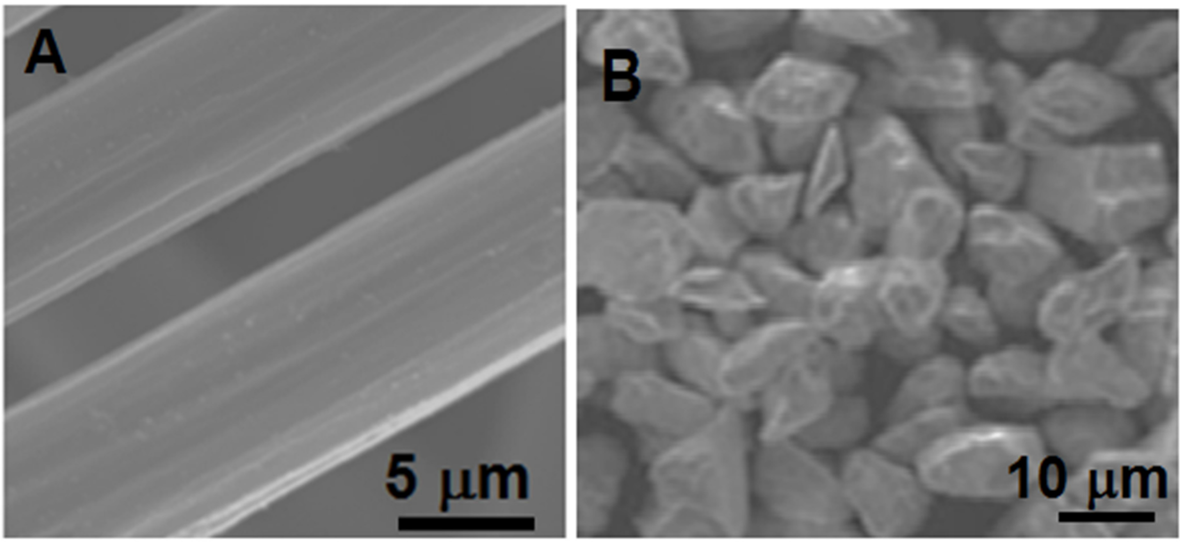

Figure S1. SEM images of (A) CF and (B) $MnCO_3$NS.

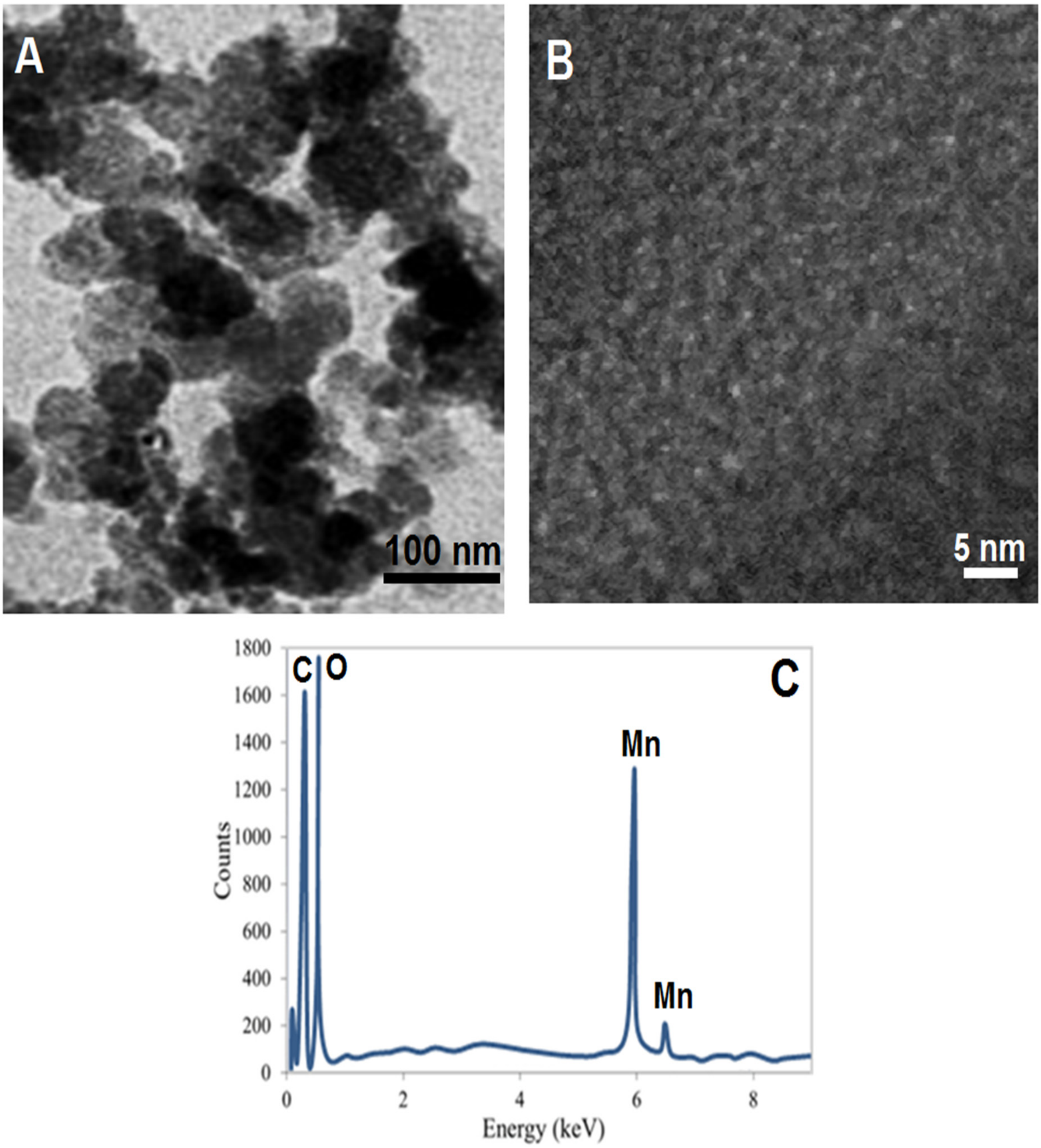

Figure S2. (A) TEM image of $MnCO_3NS$, (B) HRTEM image of $MnCO_3NS$ and (C) EDX spectrum of $MnCO_3NS/CF$ composite.

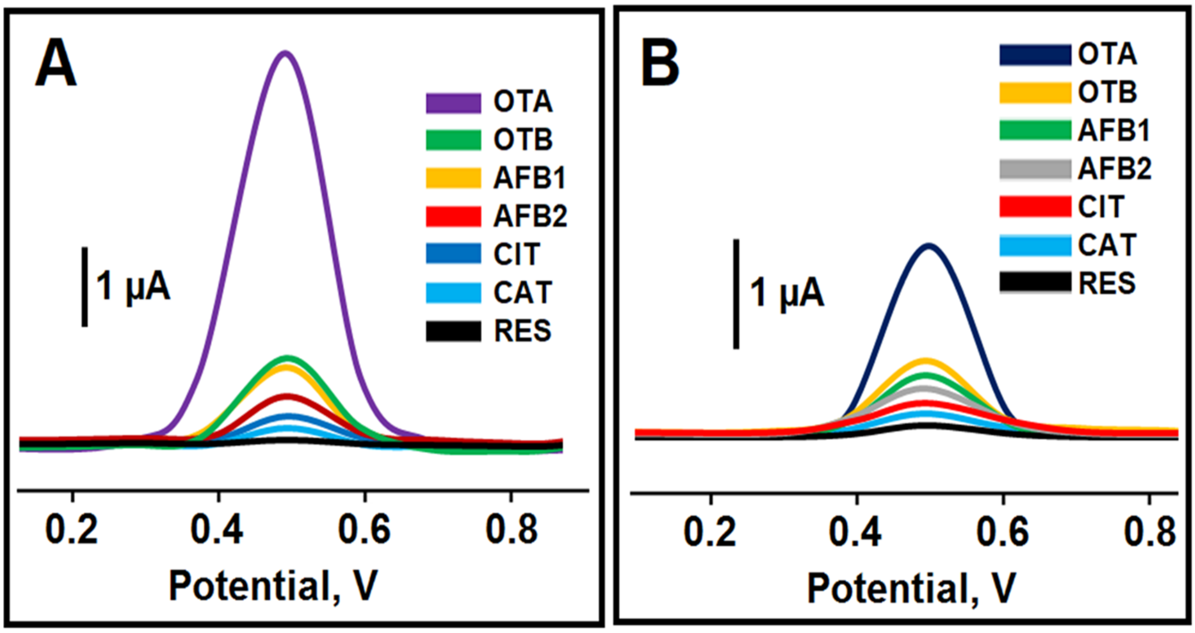

Figure S3. DPVs of (A) MIP/$MnCO_3$NS/CF/GCE and (B) NIP/$MnCO_3$NS/CF/GCE in 1.0 nmol L^{-1} OTA, 100.0 nmol L^{-1} OTB, 100.0 nmol L^{-1} AFB1, 100.0 nmol L^{-1} AFB2, 100.0 nmol L^{-1} CIT, 100.0 nmol L^{-1} CAT and 100.0 nmol L^{-1} RES.

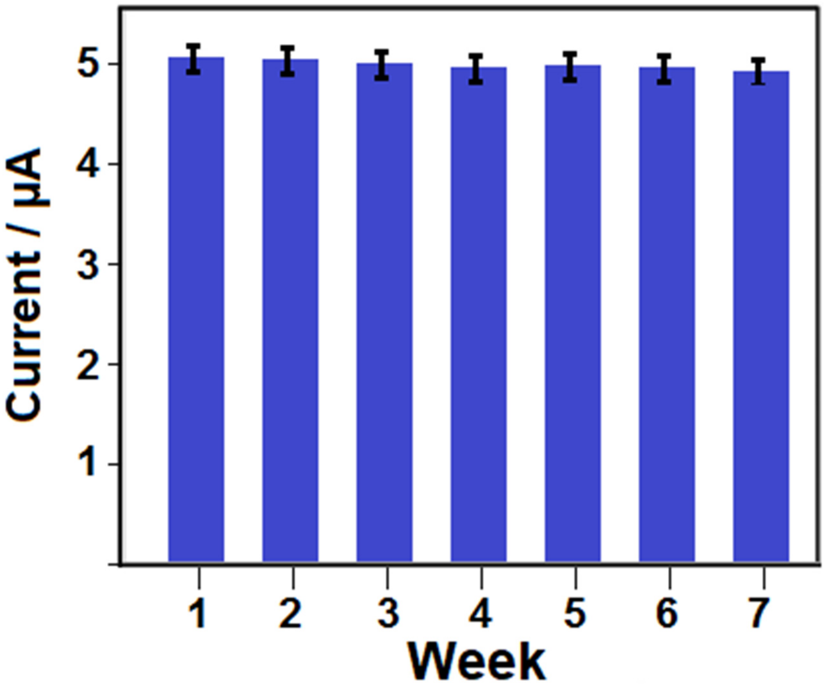

Figure S4. Stability test of MIP/$MnCO_3$NS/CF/GCE including 0.5 nmol L^{-1} OTA (n = 6).
